# NLRP3 inflammasome as a novel therapeutic target for Alzheimer’s disease

**DOI:** 10.1038/s41392-020-0145-7

**Published:** 2020-04-01

**Authors:** Yun Zhang, Zhifang Dong, Weihong Song

**Affiliations:** 1 Townsend Family Laboratories, Department of Psychiatry, The University of British Columbia, 2255 Wesbrook Mall, Vancouver, BC V6T 1Z3 Canada; 2 Pediatric Research Institute, Ministry of Education Key Laboratory of Child Development and Disorders, National Clinical Research Center for Child Health and Disorders, China International Science and Technology Cooperation Base of Child Development and Critical Disorders, Chongqing Key Laboratory of Translational Medical Research in Cognitive Development and Learning and Memory Disorders, Children’s Hospital of Chongqing Medical University, Chongqing, China

**Keywords:** Diseases of the nervous system, Neuroimmunology

In a recent publication in *Nature*, Ising et al. reported the effect of the NLRP3 inflammasome on Aβ-induced tau pathology in Alzheimer’s disease (AD). The findings indicate that NLRP3 may be a novel target for the treatment of AD.^1^

The presence of neuritic plaques and neurofibrillary tangles (NFTs) in the brain are two pathological hallmarks of AD. Neuritic plaques comprise amyloid β protein (Aβ), while NFTs are formed by hyperphosphorylated tau protein. Although the clinical symptoms and pathological features of AD are well defined, the mechanism underlying neuronal death and cognitive impairments in AD remains elusive. Several hypotheses, including the tau hypothesis,^2^ the amyloid hypothesis^3^ and the inflammation hypothesis,^4^ have been proposed to explain the pathogenesis of AD.

**Fig. 1:**
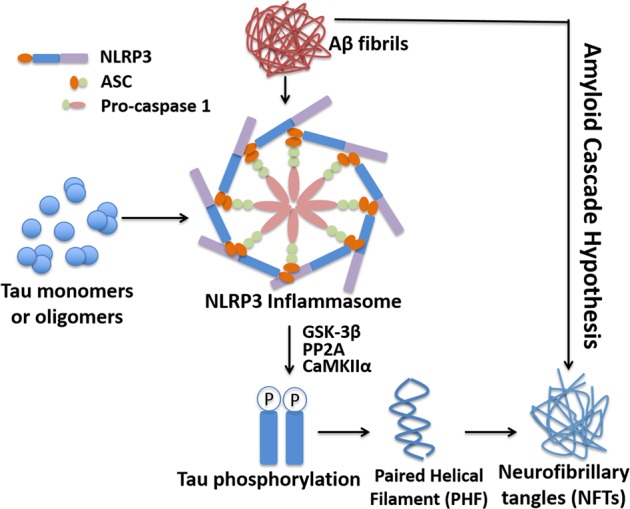
Activation of the NLRP3 inflammasome increases tau hyperphosphorylation. Tau monomers and oligomers stimulate NLRP3 activation. The loss of NLRP3 inflammasome function reduces tau pathology. Fibrillar Aβ induces tau pathology in an NLRP3-dependent manner, which further supports the amyloid cascade hypothesis

NLRP3 (NOD-, LRR- and pyrin domain-containing protein 3), or cryopyrin, is predominantly expressed in macrophages, and is encoded by the *NLRP3* gene on human chromosome 1. NLRP3 acts as a sensor molecule, and together with the adaptor protein ASC and pro-caspase-1, forms the NLRP3 inflammasome, a protein complex that is critical for the innate immune system. It induces the cleavage of cytokine precursors to generate active interleukin-1β (IL-1β) and IL-18. The NLRP3 inflammasome has been implicated in a wide range of diseases, including AD. The NLRP3 inflammasome has been shown to colocalize with neuritic plaques, and its level is substantially elevated in AD brains.^5^ The activation of the NLRP3 inflammasome enhances Aβ aggregation by reducing Aβ phagocytosis.^5,6^ However, its effect on tau pathology is not known. In a recently published article titled “NLRP3 inflammasome activation drives tau pathology”, Ising et al.^1^ reported that the inhibition of NLRP3 inflammasome activity decreased tau phosphorylation and aggregation. Tau monomers and oligomers stimulated NLRP3 activation, but tau fibrils did not. In addition, fibrillar Aβ facilitated tau pathology by activating NLRP3, indicating that the NLRP3 inflammasome may be a potential therapeutic target for AD (Fig. 1).

Aβ is generated from amyloid precursor protein (APP) through cleavage by β-secretase (BACE1) and γ-secretase.^7,8^ The widely accepted amyloid cascade hypothesis posits that Aβ aggregation is the most critical step in AD pathogenesis. Aβ aggregation acts as an initiating event, contributing to other pathological changes in AD. Aβ has been shown to activate kinases, leading to increased tau phosphorylation,^9^ while a reduction in Aβ inhibits tau neurotoxicity and ameliorates cognitive deficits in AD mouse models.^10^ Tau, a microtubule-associated protein, has six isoforms in the human brain. Tau undergoes post-translational modifications, including phosphorylation, methylation, acetylation, ubiquitination, glycation and SUMOylation. Tau binds to tubulin assemblies and stimulates polymerization to regulate the stability of these assemblies. Alterations in the post-translational modification of tau, such as an increase in hyperphosphorylation, result in the detachment of tau from microtubules, and therefore impair axonal stability and neuronal plasticity. The amyloid cascade and tau hypothesis provide a direction for the development of therapeutic treatments for AD. However, drug trials have failed due to the low specificity of the drugs or because they were applied too late to be effective.

Inflammation plays an important role in AD pathogenesis. Activated microglia and astrocytes secrete a variety of proinflammatory cytokines and toxic products, leading to neuronal dysfunction and apoptosis. Neuroinflammation also exacerbates other AD pathologies. The transcription factor NFκB is considered a primary regulator of inflammatory responses. The activation of NFκB stimulates the BACE1 cleavage of APP and Aβ production by enhancing *BACE1* expression.^7^ The inflammasome is an intracellular protein complex that regulates the maturation of IL-1β and IL-18, which are significantly increased in AD brains and associated with the onset and progression of the disease. However, the exact role of the inflammasome in AD pathogenesis and its relationship with other AD pathologies are not fully understood.

Ising et al. examined the effect of the NLRP3 inflammasome on tau pathology. They found that genetically inhibiting NLRP3 activity in Tau22/Asc^−/−^ and Tau22/Nlrp3^−/−^ mice significantly reduced tau phosphorylation in different brain regions and prevented cognitive decline in these mice. This effect was due to the regulatory effect of NLRP3 on tau kinases and phosphatases, including PP2A, GSK-3β and CaMKΙΙα. It has been shown that Aβ interacts with the NLRP3 inflammasome and contributes to its activation.^6^ Like Aβ, tau can induce the activation of the NLRP3 inflammasome in microglia, resulting in the production of mature IL-1β. Thus, the authors further investigated the tau species that are involved in the activation of the inflammasome. The effects of wild-type and P301S mutant tau proteins of different forms (monomers, oligomers and fibrils) were examined. The monomeric and oligomeric proteins markedly elevated IL-1β levels through ASC and NLRP3, while tau fibrils had no significant effect. It has been reported that tau oligomers and fibrils, but not monomers, impair neuronal activity and viability, leading to AD. However, the study demonstrated that soluble tau monomers also promoted tau pathology and neurodegeneration by activating the NLRP3 inflammasome in microglia.

The link between amyloid deposition and tangle formation is one of the key issues for AD pathogenesis and drug development. Ising et al. found that the injection of Aβ-containing APP/PS1 brain homogenates induced tau hyperphosphorylation, which is consistent with previous studies showing that Aβ aggregates act as an initiating factor in AD pathogenesis. However, the effect occurred in Tau22 mice but not Tau22/Asc^−/−^ and Tau22/Nlrp3^−/−^ mice, indicating that NLRP3 is an important mediator of Aβ-induced tau pathology. This is the first study to discover that the NLRP3 inflammasome acts as a link between Aβ plaques and neurofibrillary tangles. Aβ aggregates activate the NLRP3 inflammasome, resulting in a reduction in Aβ clearance and an increase in Aβ deposition, forming a vicious cycle.^5^ The activated NLRP3 inflammasome also promotes tau hyperphosphorylation and tangle formation. Therefore, targeting the NLRP3 inflammasome may be a promising approach for the development of therapies for AD via its effects on inhibiting both amyloid deposition and tangle formation.
